# Phenolic Antioxidant Protection in the Initial Growth of *Cryptocarya alba*: Two Different Responses against Two Invasive Fabaceae

**DOI:** 10.3390/plants12203584

**Published:** 2023-10-16

**Authors:** Lorena Rodríguez-Cerda, Lubia M. Guedes, Solange Torres, Elvis Gavilán, Narciso Aguilera

**Affiliations:** 1 Laboratorio de Semioquímica Aplicada, Departamento de Silvicultura, Facultad de Ciencias Forestales, Universidad de Concepción, Casilla 160-C, Concepción 4030000, Chile; lrodriguez2018@udec.cl (L.R.-C.); lguedes@udec.cl (L.M.G.); egavilan@udec.cl (E.G.); 2 Laboratorio de Química de Productos Naturales, Departamento de Botánica, Facultad de Ciencias Naturales y Oceanográficas, Universidad de Concepción, Casilla 160-C, Concepción 4030000, Chile; soltorres@udec.cl

**Keywords:** allelochemicals, aqueous extracts, invasive plants, *Teline monspessulana*, *Ulex europaeus*

## Abstract

The allelophatic effect of the invasive Fabaceae, *Ulex europaeus* and *Teline monspessulana*, on the production of phenolic compounds in *C. alba* seedlings was investigated. It was expected that the oxidative stress caused by the allelochemicals released by both invaders would induce a differential response in the production of phenolic compounds in *C. alba* seedlings. These antioxidant mechanisms guaranteed *C. alba* plants’ survival, even to the detriment of their initial growth. *Cryptocarya alba* seedlings were irrigated with *T. monspessulana* (TE) and *U. europaeus* (UE) extracts and water as a control. After eight months, morphometric variables were evaluated, and leaves were collected for histochemical analysis. The methanol extracts from treatments and control leaves were used for anthocyanin, phenol, and antioxidant activity quantifications. Both invasive species induced an inhibitory effect on the morphometric variables. *Teline monspessulana* induced leaf damage and increased the anthocyanin content by 4.9-fold, but did not affect the phenol content. *Ulex europaeus* induces root damage and a decrease in phenol content, but does not affect the anthocyanin content. Both Fabaceae extracts affected the profile and polyphenol concentration and consequently decreased the antioxidant capacity of *C. alba* leaves at low extract concentrations. Phenols, lignin, and ROS accumulate on *C. alba* leaves, but the histochemical reactions were less intense under UE. Although *C. alba* develops different antioxidant protection mechanisms against stress induced by UE and TE, its survival is guaranteed, even to the detriment of its initial growth.

## 1. Introduction

Biological invasions are one of the main problems causing biodiversity loss worldwide [1]. Fabaceae is one of the most invasive globally distributed plant families [2], developing numerous strategies that guarantee their colonizing success [3], such as allelopathy [4]. Bioactive compounds, mostly alkaloids and phenols, with allelochemical properties have been detected in invasive Fabaceae [5]. These allelochemicals are synthesized throughout the plant and released into the environment through leaching, biodegradation, volatilization, and exudation [6,7].

There is significant evidence that applying aqueous extracts of allelopathic plants induces oxidative stress [8], with subsequent overproduction of reactive oxygen species (ROS). ROS production as a result of allelopathy is similar to that produced during other stresses such as salinity, drought, heavy metals, extreme temperatures, and attack by pathogens and herbivores [9,10,11,12]. Different concentrations of ROS in plant cells can have different effects. For example, at low concentrations, ROS can activate plants’ defense and tolerance mechanisms against different stresses [13,14] and act as signalers that regulate the process of plant development [14,15,16]. However, high concentrations of ROS cause the oxidation of macromolecules, such as lipids, proteins, and DNA [17], leading to an imbalance between oxidant and antioxidant substances, known as oxidative stress [18]. During oxidative stress, redox homeostasis is disrupted, reducing photosynthetic yield and chlorophyll fluorescence [19,20], which involves alterations in carbohydrate metabolism and transport from leaves to roots [21]. This finally results in inhibitory modifications, sometimes irreversible, in the growth and metabolism of the acceptor plant [8,22]. Plants have mechanisms to deal with oxidative stress, though, which involve the antioxidant enzyme system and the synthesis of secondary metabolites, such as phenols [8,23].

About 700 invasive alien species have been recorded in Chile [24], of which around 72 are Fabaceae [25]. Two of the most invasive Fabaceae species in Chile are *Ulex europaeus* L. and *Teline monspessulana* (L.) K. Koch [26], both distributed between the Valparaíso Region and Los Lagos Region [3]. The spread of both invasive species has been favored by deforestation and fragmentation of the native forest, especially in the Chilean sclerophyllous forest [27]. Although the allelopathic potential of *T. monspessulana* has been little investigated, a recent study showed the presence of at least seven alkaloids and 21 phenols in *T. monspessulana* aerial organs, which were related to the allelopathic effect exerted on the native Chilean species *Nothofagus obliqua* (Mirb.) Oerst [28]. Similarly, some previous studies have demonstrated the allelopathic potential of *U. europaeus* [29,30,31]. *Ulex europaeus* was introduced intentionally in Chile for animal nutrition and to form containment fences [32,33]. This species is considered one of the 100 most invasive species in the world [34]. *Teline monspessulana* also forms dense populations, favoring the spread of forest fires [26]. 

*Cryptocarya alba* (Mol.) Looser (Lauraceae), commonly known as *peumo*, is one of the most important evergreen species of the Chilean sclerophyllous forest [35]. *Peumo* is distributed from the Coquimbo Region to the Araucanía Region [36]. However, deforestation resulting in the isolation of *C. alba* populations has led to the introduction of exotic species into their natural environments [37,38]. In contemporary south-central Chile, the presence of the invasive *T. monspessulana* and *U. europaeus* could constitute a danger to the regeneration and establishment of the native *C. alba* due to the invaders’ allelopathic effects. Because of the production of oxidative stress in the recipient species as a consequence of allelochemical stress, the antioxidant capacity of *C. alba* was evaluated under allelochemical stress induced by aqueous extracts of *T. monspessulana* and *U. europaeus*. Such allelochemical stress is expected to activate a series of reactions that trigger an antioxidant protection mechanism involving phenolic compounds to guarantee *C. alba* plants’ survival, even in detriment of their initial growth. We will also investigate whether the oxidative stress caused by the allelochemicals released by both invaders induces a differential response in the production and accumulation of phenolic compounds in *C. alba* seedlings.

## 2. Results

### 2.1. Morphometric Analysis

**Figure 1:**
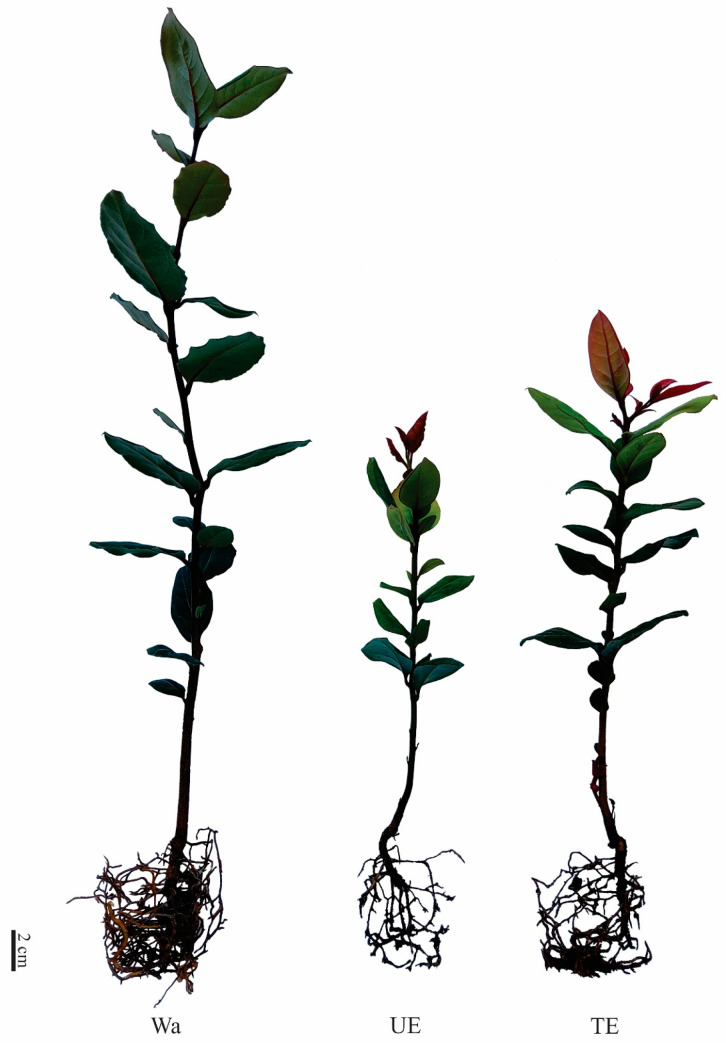
*Cryptocarya alba* seedlings after 8 months irrigated with water (Wa), *Ulex europaeus* extract (UE), and *Teline monspessulana* extract (TE).

**Figure 2:**
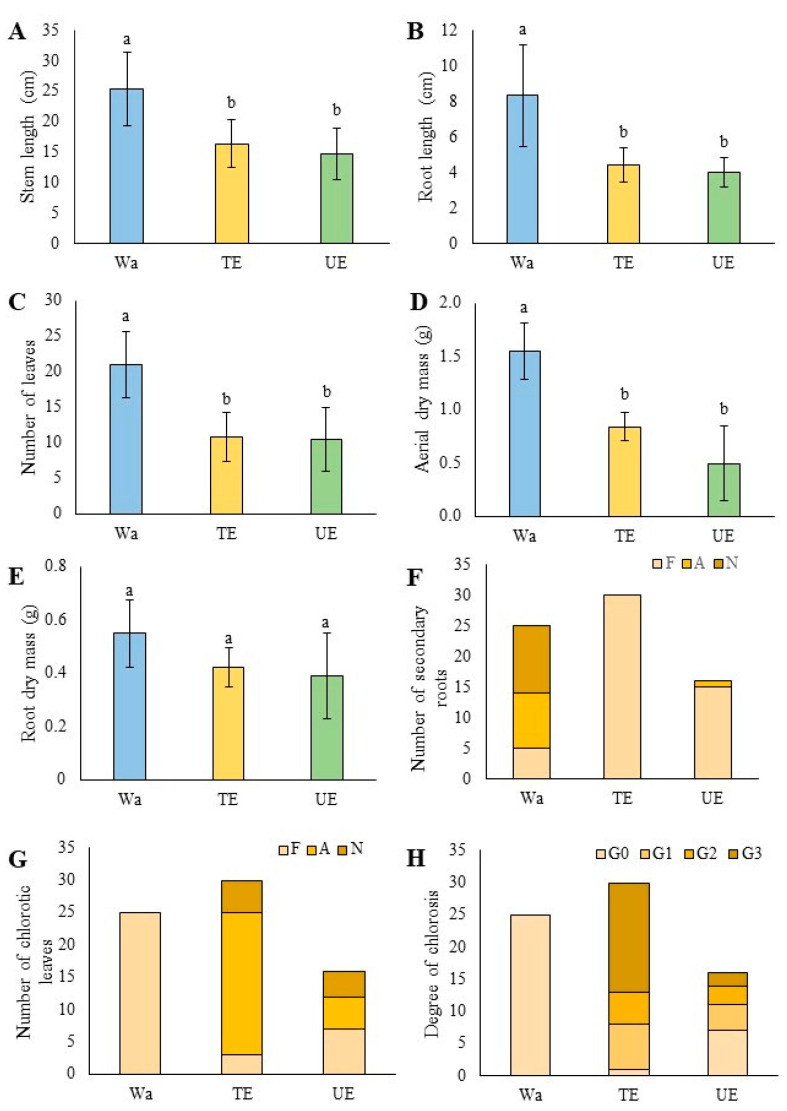
Effect of aqueous extracts of *Teline monspessulana* (TE) and *Ulex europaeus* (UE) on morphometric variables of *Cryptocarya alba* seedlings compared to control (Wa): (**A**) stem length (SL); (**B**) root length (RL); (**C**) number of leaves (NL); (**D**) aerial part dry mass (ADM); (**E**) root dry mass (RDM); (**F**) number of secondary roots (NSR) according to categories: Few (F): 0 to 12, Abundant (A): 13 to 24, Numerous (N): over 25; (**G**) number of chlorotic leaves (NCL) according to categories Few (F): 0 to 1, Abundant (A): 2 to 3, Numerous (N): over 4; (**H**) degree of chlorosis (DC) according to categories: G0, leaves without noticeable visual damage, G1, slightly chlorotic leaves, G2, severely chlorotic leaves and G3, completely damaged leaves. (**A**–**E**) are represented by their means and standard deviation; (**F**–**H**) are represented by the frequency of each category. Different letters mean significant differences between treatments for *p* ≤ 0.05.

Aqueous extracts of *T. monspessulana* (TE) and *U. europaeus* (UE) significantly affected the initial growth of *C. alba* seedlings (Figure 1). The stem (SL) and root length (RL), number of leaves (NL), and aerial dry mass (ADM) decreased under the extracts’ influence, but root dry mass (RDM) was not affected compared to the control (Wa) (Figure 2). The SL decreased significantly (*p* < 0.001), with a 36% drop in the TE treatment and 42% in the UE treatment (Figure 2A). The extract type significantly (*p* < 0.001) affected the RL, decreasing by 47% in the TE and by 52% in UE (Figure 2B). The number of leaves (NL) was also significantly affected (*p* < 0.001) according to the extract type. The TE treatment decreased the NL by 48%, while the UE treatment decreased by 50% (Figure 2C). The aerial dry mass (DM) decreased significantly (*p* < 0.001); seedlings irrigated with TE decreased by 46%, while UE treatment affected aerial biomass by 68% (Figure 2D).

The numbers of secondary roots (NSR) and chlorotic leaves (NCL) and the degree of chlorosis (DC) were also represented according to the frequency of their respective categories (Figure 2F–H). Both UE and TE treatments affect the number of secondary roots per plant, which is around 100% of plants in the few secondary roots category (Figure 2F). In seedlings irrigated with TE, an average of 6.9 secondary roots per plant were recorded, while in treatments irrigated with UE, the average number of secondary roots per plant was 6.2. The NCL increased due to extract origin since irrigation with TE and UE increased chlorotic leaf presence (Figure 2G). TE treatment also increased leaves’ chlorosis level (56.6% of the treatment presented leaves in G3), i.e., completely damaged, while in treatment irrigated with UE, only 12.5% of leaves were in G3 (Figure 2H).

### 2.2. Anthocyanin Contents

**Figure 3:**
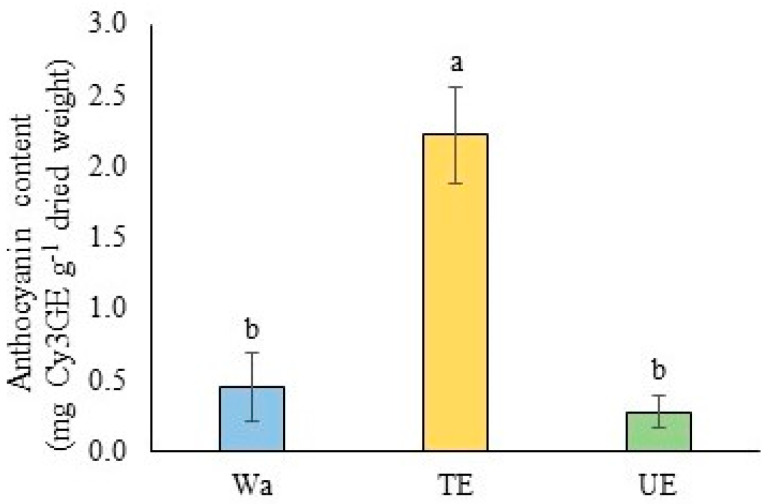
Anthocyanin content, expressed as mg Cy3GE g^−1^ dried weight, of *Cryptocarya alba* leaves irrigated with water (Wa), *Teline monspessulana* (TE) and *Ulex europaeus* (UE) extracts. Data were represented by their means and standard deviation. Different letters indicate significant differences between treatments for *p* ≤ 0.05.

Irrigation with *T. monspessulana* extract significantly increased *C. alba* leaves’ anthocyanin content (*p* < 0.001) (Figure 3). The anthocyanin content was 4.9-fold higher in the leaves irrigated with TE, while in those irrigated with UE, it decreased but without significant differences compared to the control (Figure 3).

### 2.3. Total Phenol Content and Identification

**Figure 4:**
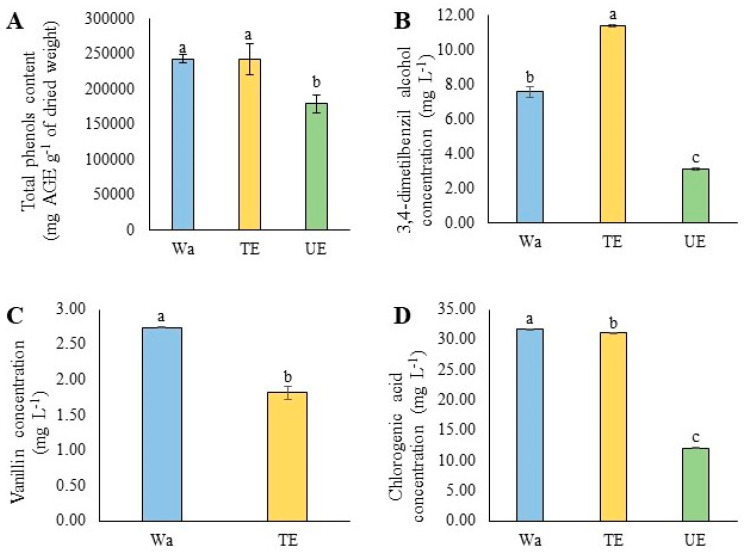
Phenol contents of *Cryptocarya alba* leaves irrigated with water (Wa), extracts of *Teline monspessulana* (TE) and *Ulex europaeus* (UE). (**A**) Total phenol content determined by Folin–Ciocalteu method; (**B**–**D**) Concentration of phenols: (**B**) 3,4-dimetilbenzil alcohol, (**C**) vanillin, and (**D**) chlorogenic acid, determined by HPLC. Data were represented by the mean and standard deviation. Different letters indicate significant differences between treatments at *p* ≤ 0.05.

The total phenol content was significantly (*p* < 0.05) affected by the treatments (Figure 4). The UE reduced *C. alba* leaves’ total phenol content by 26.1%, while TE irrigation did not significantly affect this parameter compared to the control (Figure 4). Three phenols were identified in the *C. alba* leaves: 3,4-dimethylbenzyl alcohol, vanillin, and chlorogenic acid (Figure 4B–D). However, no vanillin compound was detected in the treatment irrigated with UE. The concentration of 3,4-dimethylbenzyl alcohol was significantly affected by TE (*p* < 0.001), increasing their concentration by 50.6%, while in seedlings irrigated with UE, their concentration decreased by 58.9% compared to the control (Figure 4B). For vanillin, detected only in control leaves and TE treatment, a significant decrease (*p* < 0.001) (33.8%) was observed in the TE treatment (Figure 4C). Chlorogenic acid concentration was significantly affected by UE as well (*p* < 0.001), decreasing the concentration by 61.8%, while in seedlings irrigated with TE, the concentration was affected by 1.9% compared to the control (Figure 4D).

### 2.4. Antioxidant Activity

#### 2.4.1. DPPH Free Radical Inhibition

At higher concentrations (i.e., 0.4, 0.6, and 1.0 mg mL^−1^), the ability to eliminate the DPPH radical from *C. alba* leaves under allelochemical stress induced by TE was not affected (Table 1). However, at a concentration of 0.1 mg mL^−1^, the *C. alba* leaves subjected to both extracts (TE and UE) decreased their antioxidant capacity compared to the control (Wa), being significantly lower in the leaves under UE-induced stress, also at 0.4 mg mL^−1^ (Table 1). 

#### 2.4.2. ABTS Free Radical Inhibition

*Cryptocarya alba* leaves’ antioxidant capacity to eliminate the ABTS radical was significantly affected by the extracts’ concentrations (Table 2). Whereas at a concentration of 1 mg mL^−1^, there were no significant differences between the control (Wa) and the treatments (TE and UE), at lower concentrations (0.1–0.6 mg mL^−1^), the UE significantly decreased *C. alba* leaves’ antioxidant capacity to eliminate the ABTS radical (Table 2). However, TE did not affect this parameter when compared to the control (Table 2).

### 2.5. Histolocalization of Secondary Metabolites

**Figure 5:**
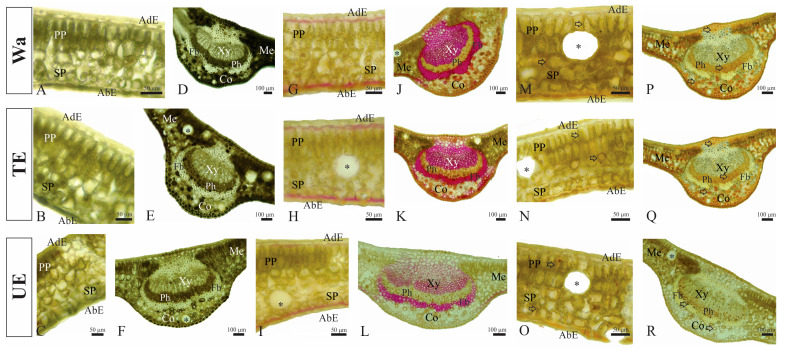
Histochemical detection of total phenols, lignin, and reactive oxygen species (ROS) of *Cryptocarya alba* leaves irrigated with water (Wa), *Teline monspessulana* (TE) and *Ulex europaeus* (UE) extracts. (**A**–**F**) Total phenols detected as black precipitates by reaction with 3% iron chloride in the mesophyll (**A**–**C**) and midrib (**D**–**F**). (**G**–**L**) Lignin detected in pink by reaction with Wiesner’s reagent in the mesophyll (**G**–**I**) and midrib (**J**–**L**). (**M**–**R**) ROS detected as brown color (black arrow) by reaction with 0.5% 3,3′-diaminobenzidine in the mesophyll (**M**–**O**) and midrib (**P**–**R**). Abbreviation: AbE, abaxial epidermis; AdE, adaxial epidermis; Co, collenchyma; Fb, fibers; Me, mesophyll; Ph, phloem; PP, parenchyma palisade; PS, parenchyma spongy; Xy, xylem. The black asterisk indicated a secretory duct.

Regardless of the treatment (TE and UE), phenols, lignin, and ROS accumulate on *C. alba* leaves (Figure 5). The reaction with 3% iron chloride for total phenols had a weaker process in the mesophyll of the control leaves and the two treatments (Figure 5A–C) than in the midrib (Figure 5D–F). Total phenols accumulated in palisade and spongy parenchyma cells and in the cytoplasm of abaxial epidermis cells of control leaves (Figure 5A) and were irrigated with TE (Figure 5B) and UE (Figure 5C). The reaction was weaker in the latter one. In the midrib of control and TE-irrigated leaves, phenols accumulated intensely in the cell cytoplasm of both epidermis, collenchyma, and phloem parenchyma (Figure 5D,E), while in UE-irrigated leaves, phenols were weakly detected in the cytoplasm of both epidermis cells and in some collenchyma cell walls (Figure 5F).

For lignin detection, the reaction with Wiesner’s reagent indicated the presence of this metabolite in the inner cell walls of both epidermis in the control and treatment leaves (Figure 5G–I). The reaction was more intense in control leaves and less intense in leaves irrigated with UE (Figure 5I). Adaxial collenchyma cell walls, some abaxial collenchyma cells, and the perivascular fibers of the control leaf midrib are strongly lignified (Figure 5J–L). On leaves irrigated with TE, lignin accumulated at the same sites as control leaves, but the reaction was less intense (Figure 5H,K). In *C. alba* leaves with UE, lignin was weakly deposited in the adaxial collenchyma and vascular fiber cell walls (Figure 5L).

Hydrogen peroxide was detected in the palisade cell walls and in the apoplast of spongy cells among control and TE-irrigated leaves (Figure 5M–O). When *C. alba* leaves were irrigated with UE, ROS accumulated in palisade cell walls, while in spongy cells they were weakly detected (Figure 5O). In the midrib of control and TE-irrigated leaves (Figure 5Q), ROS were detected in the cytoplasm of collenchyma cells and phloem parenchyma. In UE-irrigated leaves, the phloem parenchyma cytoplasm accumulated ROS, while in the collenchyma cells, they accumulated in the cell walls (Figure 5R).

## 3. Discussion

Current results indicate that *T. monspessulana* and *U. europaeus* affect *C. alba* seedlings’ early growth. The aqueous extracts of both invasive species induced an inhibitory effect on the morphometric variables of *C. alba*. However, the *T. monspessulana* extract induced a high number of severely damaged chlorotic leaves, while the *U. europaeus* extract induces damage at the root level with less root system development, a shorter main root length, and fewer secondary roots. The leaf damage induced by TE could be due to prolonged allelopathic stress exposure. Under these conditions, proteases are activated, inducing programmed cell death [17]. This implies that *C. alba* sacrifices the leaves so the rest of the plant can survive, reflecting a delay in growth and development. It is well documented that allelopathic stress can also affect the structure and activity of the root apical meristem, which affects water and nutrient absorption, implying a delay in root growth [39,40,41], as has also been observed in *C. alba* seedlings under UE stress. Prior works show the allelopathic capacity of *T. monspessulana* and *U. europaeus* over the early growth of *N. obliqua* [28] and *Quillaja saponaria* Molina [42], two Chilean native species. The allelopathic effect of *U. europaeus* on the growth and development of agricultural species [30], *Lactuca sativa* L. [31], and *Amaranthus retroflexus* L. [29], has also been shown. However, the allelopathic capacity of *T. monspessulana* has been little studied. 

The phytotoxic effect of *T. monspessulana* and *U. europaeus* on *C. alba* seedlings could be related to the high phytotoxic features of several Fabaceae species [43], mainly because they synthesize quinolizidine alkaloids and phenols [44]. For example, the presence of alkaloids N-methylcytisine, lupanine, argentamine, thermopsine, N-[2-aminoethyl] cytisine, and cytisine in the methanol extract of *U. europaeus* has been tied to cytotoxic effects [31]. The alkaloids caulophylline, lupanine, aphylline, anagyrine, sophocarpine, ellipticine, and cytisine have also been detected in *T. monspessulana* [28]. Allelopathic compounds are released into the environment through leaching, volatilization, root exudation, and organ decomposition [6]. These allelopathic compounds induce oxidative stress and ROS overproduction [45], alter cell membrane structure and function, and degrade chlorophyll, affecting the photosynthesis process [19,46]. Phytotoxic compounds can also affect nutrient uptake [47], induce root necrosis [5], and induce root cell membrane alterations [48], altering the recipient plants’ early growth. The photosynthetic process as well as the nutrient uptake are likely affected by the extracts of the invasive Fabaceae, but TE affects more at the foliar level, while UE affects the root level. This assumption should be corroborated with structural and photosynthetic performance in future studies.

### Different Mechanisms of Native C. alba to Deal with Allelochemical Stress

*Cryptocarya alba* has high antioxidant potential, mainly due to the presence of phenolic compounds in its organs [49]. The phenolic compounds detected here, 3,4-dimethylbenzyl alcohol, vanillin, and chlorogenic acid, were previously reported in *C. alba* [50,51,52,53]. However, allelochemical stress induced by *T. monspessulana* and *U. europaeus* modifies the *C. alba* phenolic profile, although each extract induces different response mechanisms. 

Under *T. monspessulana* stress, *C. alba* leaves maintain their antioxidant potential, probably because the total phenol content is not affected, although there was an increase. 

In the synthesis of 3,4-dimethylbenzyl alcohol and anthocyanin. The current results agree with some previous studies. For example, in radish seedlings subjected to aqueous extracts of peppermint, no changes were observed in the antioxidant enzyme system, but an increase in the total phenolic content was observed [54]. Similarly, in *Lactuca sativa* L. plants under allelopathic stress from some Cupressacea species, negative effects were recorded on initial growth and chlorophyll content, but an increase in the concentration of flavonoids and tannins in the recipient plant was also observed [55]. For both study systems, the authors suggest that phenol accumulation is an antioxidant mechanism in response to allelopathic stress, acting as the main ROS dissipative mechanism. 

Allelopathic stress induced by TE also triggers the synthesis of high anthocyanin concentrations, probably due to TE-induced leaf damage. Anthocyanin synthesis in the leaves has been associated with chlorophyll decomposition due to different stresses [56,57], which act as scavengers of free radicals [58,59,60]. The high concentrations of 3,4-dimethylbenzyl alcohol in *C. alba* seedlings under TE stress could also act as a ROS remover. This alcohol is reported as a potent antioxidant [61] in species such as *Dillenia suffruticosa* (Griff.) Martelli [62], *Syringa vulgaris* L. [63], *Glycine max* L., and *Vigna radiata* L. [64]. Without invalidating other enzymatic and non-enzymatic antioxidant mechanisms, the maintenance of phenolic content and the high anthocyanin and 3,4-dimethylbenzyl alcohol concentrations could act synergistically as ROS scavenger mechanisms in *C. alba* seedlings under TE stress.

*Ulex europaeus* inhibits the antioxidant potential of *C. alba* leaves due to lowered total phenol content, as well as by inhibiting vanillin synthesis and decreasing chlorogenic acid and 3,4-dimethylbenzyl alcohol concentrations. *Ulex europaeus* probably triggers other antioxidant mechanisms, such as enzymatic mechanisms, which we unfortunately did not evaluate. The root system is the first organ impacted by the discharge of allelochemicals into the soil [41]. Root growth inhibition is the mode of action for most allelochemicals described to date [8]. Allelochemicals stop or decrease cell division and induce lignification of the cell walls of the root apex, mainly xylem cells, preventing normal root growth and changes in their ultrastructure [8,9], as previously reported for the native *Q. saponaria* under allelopathic stress induced by *Acacia dealbata* Link (Fabaceae) [48].

The histochemical results also corroborate the quantitative results. Phenols and ROS were detected more intensely and in more cell types in leaves irrigated with TE. Both phenols and ROS co-occur in the same cellular compartments, mainly in the spongy and phloem parenchyma. The co-occurrence of phenolic compounds and ROS has been described in other biotic stresses [65], corroborating phenols’ role in ROS removal. Histochemical tests also showed less intense reactions and fewer lignin deposition sites in *C. alba* leaves under *U. europaeus* stress, contrary to plants observed under TE. This result confirms the assumption that TE causes greater damage at the foliar level, responding with a higher degree of lignification. Lignin synthesis is a response to conditions that cause biotic or abiotic stress. ROS favors cell wall lignification, which stimulates dehydrogenation, with coupling acting as ROS scavengers [65]. Regardless of the antioxidant mechanism triggered, it is not sufficient to remove the ROS produced, retarding the growth of *C. alba* seedlings under the stress of both invaders. 

## 4. Materials and Methods

### 4.1. Sampling Site and Sample Collection

*Cryptocarya alba* and *T. monspessulana* were collected on the Concepción campus, Universidad de Concepción (36°50′09.4″ S 73°01′49.9″ W), Chile. *Ulex europaeus* were collected at Caleta Lenga, Hualpén peninsula (36°45′59.097″ S 73°10′23.416″ W), Chile. Seeds and soil of *C. alba* were collected under tree canopies at five different points. Seeds were air-dried and stored at 4 °C. The aerial organs of *U. europaeus* and *T. monspessulana* were cut into small pieces and macerated in distilled water (250 g in 700 mL of water) with shaking at 200 rpm in an orbital sieve (DLAB SK-L330-Pro, China). After three days, the extracts were filtered and stored at 4 °C in darkness. Each extract was mixed with 2 mL L^−1^ of plant preservative mixture (PPM) to prevent microorganism growth. These aqueous extracts were used for seedling irrigation.

### 4.2. Soil Assay Establishment

*Cryptocarya alba* soil was deposited into three plastic trays of 50 alveoli (50 × 46 × 30 mm) (n = 3), with three seeds sown in each alveolus. After germination and seedling emergence, one tray was assigned as the control and irrigated with tap water (Wa), another tray was irrigated with aqueous extract of *T. monspessulana* (TE), and the third tray was irrigated with aqueous extract of *U. europaeus* (UE). In each alveolus, a single seedling was left, and the rest were transplanted to the alveoli where germination had not occurred. Transplanting was only carried out before starting the irrigation with the extracts and after the first week of transplanting. Irrigation was carried out every 3 or 4 days at a rate of 50 mL of water or extract per alveolus. The trays were randomly placed in a growth chamber (22 °C temperature, 60–65% relative humidity, light intensity of 50 µmol m^−2^ s^−1^, and 16 h light/8 h dark photoperiod).

### 4.3. Morphometric Analysis

Morphometric analysis was performed on all *C. alba* seedlings from the control (Wa) and treatments (TE and UE). The SL, RL, NL, NSR, NCL, and DC were measured within eight months of the assay establishment. The NSR and NCL variables were divided into three categories: Few (F), abundant (A), and numerous (N) (Figure 2F,G). The NSR categories were established according to the following ranges: F: 0–12 secondary roots; A: 13–24 secondary roots; N: 25–36 secondary roots. NCL categories were established according to the following ranges: F: 0–1 chlorotic leaf; A: 2–3 chlorotic leaves; N: 4–5 chlorotic leaves. The degree of chlorosis (DC) was established according to the following categories: (G0) leaves without noticeable visual damage, (G1) slightly chlorotic leaves, (G2) severely chlorotic leaves, and (G3) completely damaged leaves. Seven seedlings were also selected from the Wa and TE treatments, and six seedlings from the UE treatment for the measurements of aerial (ADM) and root dry mass (RDM). In this process, seedlings (aerial organs and roots separately) were dried at 60 °C for 48 h in a forced air-drying oven (Venticel 111 Eco, Múnich, Germany) and weighed on a precision balance (RADWAG 2/A2, Radom, Poland).

### 4.4. Chemicals and Reagents

Folin–Ciocalteu’s phenol reagent, methanol, ethanol, glacial acetic acid, formalin, potassium chloride, sodium acetate, sodium carbonate (Na_2_CO_3_), potassium persulfate, hydrochloric acid, and ferric(III) chloride were purchased from Merck (Darmstadt, Germany). The diammonium salt of 2,2-azinobis(3-ethylbenzothiazoline-6-sulfonic acid (ABTS), 6-hydroxy-2,5,7,8-tetramethylchromane-2-carboxylic acid (Trolox), gallic acid and 2,2-diphenyl-1-picrylhydrazyl (DPPH), 3,3′-diaminobenzidine (DAB), p-hydroxybenzoic acid, vanillic acid, 3,4-dimethoxyphenol, chlorogenic acid, floroglucinol, quercetin 3-rutinoside, and quercetin were purchased from Sigma-Aldrich (Saint Louis, MO, USA).

### 4.5. Plant Material Processing

*Cryptocarya alba* leaves were collected from the control (Wa) and each treatment (TE and UE) for spectrophotometric analysis. Leaves (n = 7 per treatment and control) were frozen in liquid nitrogen and stored at −23 °C. For histochemical analyses, seven leaves (n = 7 per treatment and control) at the second node were fixed in FAA (37% formalin, glacial acetic acid, and 70% ethanol) [66] for 48 h and subsequently stored in 70% ethanol.

### 4.6. Spectrophotometric Analyses

The leaves previously stored at −23 °C were freeze-dried (Lyophilizer Crydos, Telstar, Mexico City, Mexico), homogenized at 1500 rpm (MiniG^®^ 1600, Metuchen, NJ, USA), and weighed separately according to each spectrophotometric analysis. The absorbance of each analysis was measured in triplicate in a microplate reader (ELX800, BioTek, Santa Clara, CA, USA).

### 4.7. Anthocyanin Extraction and Quantification

To extract anthocyanin, 1 g of freeze-dried *C. alba* leaves (control and treatments) was put in 10 mL of acidified 80% methanol solution [67], sonified in an ultrasonic bath, and centrifuged at 3500 rpm for 5 min. A supernatant fraction was collected and mixed with a potassium chloride buffer solution, pH 1.0, and another fraction with a sodium acetate buffer, pH 4.5. Anthocyanin quantification was performed by the differential pH method proposed by [68]. Absorbances were read at 530 nm and 700 nm. The results were expressed as mg of cyanidin-3-glucoside per g of dried weight (mg Cy3GE g^−1^ dried weight).

### 4.8. Preparation of Methanol Extracts for Phenol Quantification, Identification and Antioxidant Activity

Methanol extracts were prepared from *C. alba* leaves of control and treatments, previously freeze-dried and homogenized. For this, 1.5 g of a pool of *C. alba* leaves from each treatment (ET and EU) and control (Wa) was macerated in 15 mL of 100% methanol [65]. Extraction was performed three times with the same volume of methanol. The extracts were pooled, filtered, and dried at 37 °C under reduced pressure on a rotary evaporator (LabTech, Sorisole, Italy). 

### 4.9. Quantification and Identification of Total Phenols

Total phenol content was determined by the Folin–Ciocalteu method according to the methodology described by Mongkolsilp et al. (2004) [69]. For this, 10 µL of each extract (0.1 mg mL^−1^) was mixed with 20 µL of Folin–Ciocalteu’s reagent and 200 µL of distilled water. After 5 min, 100 µL of 15% Na_2_CO_3_ was added, and the reaction was incubated in darkness for 1 h. Absorbance was measured at 750 nm. A standard solution of gallic acid was used (0.625–200 µg mL^−1^) to generate a calibration curve. Total phenol content was expressed as mg of gallic acid equivalents per gram of dry sample (mg GAE g^−1^ of dried weight).

The phenolic profile was determined using a high-performance liquid chromatograph (HPLC) coupled to a Hitachi Primaide HPLC-DAD diode array detector, equipped with a Kromasil^®^ C18 column. The two mobile-phase solvents used were 1% formic acid in water and acetonitrile. The separation was performed at a flow rate of 1 mL min^−1^, and the injection volume used was 10 µL. The detector was set at 250, 280, 320, and 360 nm wavelengths. The compounds’ concentrations were determined from a calibration curve with high purity standards: p-hydroxybenzoic acid, vanillic acid, 3,4-dimethoxyphenol, gallic acid, chlorogenic acid, quercetin 3-rutinoside, and quercetin. Each sample was read in triplicate.

### 4.10. Antioxidant Activity

#### 4.10.1. Inhibition of the DPPH^+^ Radical

The ability of the methanol extracts from the control (Wa) and treatments (TE and UE) to scavenge the DPPH radical was determined using the methodology described by Singh et al. (2016) [70]. The reaction was performed by mixing 180 µL of the DPPH radical with 20 µL of the sample extracts at different concentrations (0.1–1 mg mL^−1^). A methanol solution of Trolox (0.1–10 mg mL^−1^) and a blank (DPPH radical without samples) were used as controls. Sample and control absorbances were read at 515 nm after 30 min in the dark. 

#### 4.10.2. Inhibition of ABTS^•+^ Radical

The capacity of the methanol extracts from the control (Wa) and treatments (TE and UE) to scavenge the ABTS radical was performed as described by Re et al. (1999) [71]. To activate the radical, the chemical reaction of ABTS (38.8 mg in 20 mL of distilled water) with potassium persulfate (K_2_S_2_O_8_) (6.6 mg in 20 mL of distilled water) had to take place for 16 h. The ABTS radical cation (ABTS^•+^) was dissolved in water until obtaining an absorbance of 0.70 nm at 750 nm. To evaluate the inhibition of the radical, 180 µL of ABTS^•+^ was mixed with 20 µL of each sample at different concentrations (0.1–1 mg mL^−1^). After 25 min in darkness, the absorbance was read at 750 nm. The Trolox solution and a blank (ABTS^•+^ without samples) were used as a control.

The capacity to scavenge DPPH^+^ and ABTS^•+^ radicals was calculated and expressed as inhibition percentage using the following equation:

% Inhibition of radical: (*Ac − As*)/*Ac* × 100;

where *Ac* is control absorbance and *As* is sample absorbance.

### 4.11. Histochemical Analysis

The control (Wa) and treatment (TE and UE) leaves (n = 5 for control and each treatment) were freehanded and subjected to histochemical tests for total phenols, lignin, and ROS (H_2_O_2_) detection. For total phenols, the sections were reacted with 3% iron chloride, with the black precipitate indicating a positive reaction [66]. To detect lignin, Wiesner’s reagent was used; the pink coloration indicated lignin deposition [72]. Hydrogen peroxide was detected in brown by reaction with 0.5% DAB [73]. Unstained sections were used for comparison. Histochemical reactions were observed and photographed with a Leica photomicroscope (Leica DM2500, Wetzlar, Germany).

### 4.12. Statistical Analysis

Data normality assumption testing was performed with the Shapiro–Wilk test, and homoscedasticity testing was performed with Bartlett’s test. Data were analyzed under one-way ANOVA to determine significance, and Tukey’s test was applied to compare means. For the MRL variable, Welch ANOVA and the Games–Howell test were applied, with Student’s *t*-test used to analyze the vanillin concentration. Statistical analyses were performed with RStudio 4.2.1 software with a significance level of 0.05.

## 5. Conclusions

The allelochemicals released by *T. mospessulana* act mainly on leaf tissues. At this level, the total phenolic content is not affected, but an increase in the anthocyanin and 3,4-dimethylbenzyl alcohol concentrations is induced, which act as ROS scavengers. This assumption was corroborated by the high antioxidant power of *C. alba* leaves and the co-occurrence of phenols and ROS in the same cellular compartments under *T. mospessulana* extract. However, the allelochemicals released by *U. europaeus* act mostly on the *C. alba* root systems. These allelochemicals inhibit antioxidant mechanisms involving phenolic compounds, which was also confirmed histochemically. Regardless of this differential response induced by *T. mospessulana* and *U. europaeus* extracts, both species retard early *C. alba* growth.

## Figures and Tables

**Table 1:** DPPH free radical scavenging activity of *Cryptocarya alba* leaves, previously irrigated with water (Wa), *Teline monspessulana* (TE) and *Ulex europaeus* (UE) aqueous extracts. Results appear as percentage inhibition of the radical. Data were represented by the mean ± standard deviation. Different letters in the same row indicate significant differences between treatments at *p* ≤ 0.05.

Concentration (mg mL^−1^)	DPPH Method			
	Trolox	Wa	TE	UE
1.0	84.33 ± 0.51 ^a^	80.10 ± 1.79 ^b^	80.08 ± 1.22 ^b^	80.53 ± 0.33 ^b^
0.6	84.27 ± 0.17 ^a^	82.31 ± 0.49 ^b^	81.75 ± 0.16 ^b^	81.66 ± 0.16 ^b^
0.4	84.77 ± 0.33 ^a^	82.15 ± 0.33 ^b^	82.23 ± 0.32 ^b^	81.34 ± 0.16 ^c^
0.1	84.77 ± 0.33 ^a^	49.05 ± 0.97 ^b^	43.63 ± 1.45 ^c^	32.82 ± 0.65 ^d^

**Table 2:** ABTS free radical scavenging activity of *Cryptocarya alba* leaves, previously irrigated with water (Wa), *Teline monspessulana* (TE) and *Ulex europaeus* (UE) aqueous extracts. Results appear as percentage inhibition of the radical. Data were represented by the mean ± standard deviation. Different letters in the same row indicate significant differences between treatments at *p* ≤ 0.05.

Concentration (mg mL^−1^)	ABTS Method			
	Trolox	Wa	TE	UE
1.0	94.06 ± 0.07 ^a^	93.18 ± 0.52 ^b^	93.16 ± 0.22 ^b^	92.85 ± 0.30 ^b^
0.6	93.84 ± 0.15 ^a^	92.10 ± 0.51 ^b^	92.72 ± 0.31 ^b^	90.48 ± 0.22 ^c^
0.4	93.79 ± 0.22 ^a^	89.17 ± 0.80 ^b^	89.25 ± 1.88 ^b^	76.84 ± 1.09 ^c^
0.1	77.79 ± 0.22 ^a^	35.94 ± 0.80 ^b^	35.64 ± 0.89 ^b^	24.73 ± 0.38 ^c^

## Data Availability

The data presented in this study are available on request from the corresponding author.

## Abbreviations

ABTS: 2,2-azinobis-3-ethylbenzothiazoline-6-sulfonic acid; DC: degree of chlorosis DC; DPPH: 2,2-diphenyl-1-picrylhydrazyl; NL: number of leaves; NSR: number of secondary roots; NCL: number of chlorotic leaves; RL: root length; SL: stem length; TE: irrigation with aqueous extract of *Teline monspessulana*; UE: irrigation with aqueous extract of *Ulex europaeus*; Wa: irrigation with water.
